# Why do people use electronic nicotine delivery systems (electronic cigarettes)? A content analysis of Twitter, 2012-2015

**DOI:** 10.1371/journal.pone.0170702

**Published:** 2017-03-01

**Authors:** John W. Ayers, Eric C. Leas, Jon-Patrick Allem, Adrian Benton, Mark Dredze, Benjamin M. Althouse, Tess B. Cruz, Jennifer B. Unger

**Affiliations:** 1 Graduate School of Public Health, San Diego State University, San Diego, California, United States of America; 2 University of California San Diego School of Medicine, San Diego, California, United States of America; 3 Keck School of Medicine, University of Southern California, Los Angeles, California, United States of America; 4 Department of Computer Science, Johns Hopkins University, Baltimore, Maryland, United States of America; 5 Human Language Technology Center of Excellence, Johns Hopkins University, Baltimore, Maryland, United States of America; 6 Bloomberg LP, New York, New York, United States of America; 7 Institute for Disease Modeling, Bellevue, Washington, United States of America; 8 Santa Fe Institute, Santa Fe, New Mexico, United States of America; 9 New Mexico State University, Las Cruces, New Mexico, United States of America; New York City Department of Health and Mental Hygiene, UNITED STATES

## Abstract

The reasons for using electronic nicotine delivery systems (ENDS) are poorly understood and are primarily documented by expensive cross-sectional surveys that use preconceived close-ended response options rather than allowing respondents to use their own words. We passively identify the reasons for using ENDS longitudinally from a content analysis of public postings on Twitter. All English language public tweets including several ENDS terms (*e*.*g*., “e-cigarette” or “vape”) were captured from the Twitter data stream during 2012 and 2015. After excluding spam, advertisements, and retweets, posts indicating a rationale for vaping were retained. The specific reasons for vaping were then inferred based on a supervised content analysis using annotators from Amazon’s Mechanical Turk. During 2012 quitting combustibles was the most cited reason for using ENDS with 43% (95%CI 39–48) of all reason-related tweets cited quitting combustibles, *e*.*g*., *“I couldn’t quit till I tried ecigs*,” eclipsing the second most cited reason by more than double. Other frequently cited reasons in 2012 included ENDS’s social image (21%; 95%CI 18–25), use indoors (14%; 95%CI 11–17), flavors (14%; 95%CI 11–17), safety relative to combustibles (9%; 95%CI 7–11), cost (3%; 95%CI 2–5) and favorable odor (2%; 95%CI 1–3). By 2015 the reasons for using ENDS cited on Twitter had shifted. Both quitting combustibles and use indoors significantly declined in mentions to 29% (95%CI 24–33) and 12% (95%CI 9–16), respectively. At the same time, social image increased to 37% (95%CI 32–43) and lack of odor increased to 5% (95%CI 2–5), the former leading all cited reasons in 2015. Our data suggest the reasons people vape are shifting away from cessation and toward social image. The data also show how the ENDS market is responsive to a changing policy landscape. For instance, smoking indoors was less frequently cited in 2015 as indoor smoking restrictions became more common. Because the data and analytic approach are scalable, adoption of our strategies in the field can inform follow-up survey-based surveillance (so the right questions are asked), interventions, and policies for ENDS.

## Introduction

Despite the popularity of electronic cigarettes or electronic nicotine delivery systems (ENDS) [1,2], there is surprisingly little actionable intelligence on why people vape [3]. The most cited reasons include curiosity, enjoyment, and other idiopathic reasons [4–6] that do not inform any particular intervention or policy response. Fewer studies point to actionable reasons that may guide specific control measures to curtail use, such as use indoors [7] which can be mitigated by banning vaping indoors.

This knowledge gap, in part, represents the limitations of tobacco control surveillance. All of the known reasons for vaping were derived from cross-sectional surveys. For example, the International Tobacco Control Four-Country Survey asked vapers about why they use ENDS with four yes/no response items [8] Surveys require substantial time and resources to implement, meaning many reasons are never asked. Moreover, where studies rely on closed-ended response options (rather than allowing participants to use their own words) some reasons are potentially still unknown.

Supplemental approaches are needed to inexpensively and rapidly discover the reasons for using ENDS to inform the science around ENDS (such as follow-up survey-based surveillance), inform the development of policy-based control measures, or aid the design of health interventions. Herein we demonstrate the feasibility of a data-driven protocol that allows the public to describe why they vape in their own words by passively monitoring public tweets—a promising [9] but underutilized approach in public health [10–16]. Doing so lays the groundwork for a new perspective that in practice can help capture the reasons people vape quickly, informing follow-up surveillance and public health practice.

## Materials and methods

The data consisted of 3.3 million public tweets from 2012 and 2015 that was collected from the Twitter API by searching for the following ENDS-related keywords: electronic cigarette(s), electronic cig(s), e cig(s), e-cig(s), eking(s), e cigarette(s), e-cigarette(s), ecigarette(s), vape(s), vaper(s), and vaping. This data collection therefore includes all tweets about ENDS as long as they included the aforementioned terms. We then used a two-stage strategy to identify a subsample for analysis by (a) selecting organic English-language tweets that referenced ENDS use and then (b) using supervised content analysis to discover the reasons for using ENDS from these tweets.

In the first stage, irrelevant tweets were excluded by purging non-English language tweets (using the Lui method [17]) tweets with URLS (which were almost exclusively advertisements), spam, and retweets (so each tweet counted once). We identified spam tweets using a statistical machine learning classifier developed using a set of 10,157 e-cigarette tweets (60% train, 20% dev, 20% test) that were identified by Amazon Mechanical Turk annotators (mturk.com/mturk/welcome) as either spam or not spam. We used a logistic regression model to predict labels, with a set of n-gram features augmented by 300-dimensional word2vec embeddings using the Mikolov strategy [18]. Drawing from this refined database we selected tweets that indicated use ENDS by the tweeter or another person using human coders from Amazon’s Mechanical Turk [19]. Coders searched for retained tweets about ENDS use until a target sample of 2,900 for each year was achieved (thereby yielding a margin of error less than 0.02 in subsequent analysis) [20]. In practice this collective strategy retained tweets such as “*I have an electronic cig and it’s helping me quit*,” and excluded tweets such as *“Closing sale*! *#vapeporn #eking #vaping” “just read this eking article [link]*,*” or “I just saw someone vaping”* because they are advertising, included a URL, or do not indicate use, respectively. The inter-rater agreement between the Mturk coders identifying tweets as being about ENDS use was Cohen’s Kappa = 0.56, percentage agreement = 0.83. Tweets with conflicting labels were assumed to not be about ENDS use and discarded from further analysis.

In the second stage, we identified any “reasons” for use [21]. The investigators (JWA, ECL, AB, and MD) reviewed tweets and discussed reasons for vaping that emerged in the data simultaneously developing a framework and codebook for annotating the tweets. An open-ended framework was selected that allowed each tweet to have zero or multiple associated reasons; *e*.*g*., “I like ecigs because they’re cheap and taste great” would indicate lower price and flavor as reasons (Table 1). Implied reasons were also considered, e.g., “vaping in the club” would indicate vaping indoors. We then developed a protocol for the most commonly cited reasons, avoiding rare reasons because they are not high priorities for intervention or reasons focused on personal emotions because they may not inform precise interventions. The investigators then tested this protocol until a final document was agreed on. Ultimately, our data-driven pilot protocol defined 7 categories: cost, flavors, odor, safety relative to combustibles, social image, quitting combustibles, and use indoors. This protocol was then applied using Amazon’s Mechanical Turk (Cohen’s Kappa = 0.54 averaged over all 7 reasons; percentage agreement = 0.92). The results for the 7 reasons as the percent of reason-related tweets by year were described using bootstrapped 95% confidence intervals (to assess confidence in the point estimates) and non-parametric chi-squared tests (to compare differences in reasons across years).

**Table 1:** A taxonomy of reasons for vaping as reported on Twitter.

*Example Tweet*	Low Cost	Flavor Choices	Safe to Use	Can Vape Indoors	Favorable Odor	Quitting Combustibles	Social Image
I like ecigs because they’re cheap and taste great	**X**	**X**					
Love vaping in the club, sure beats freezing outside				**X**			
My ecig smells so good and its totally safe			**X**		**X**		
My ecig helped me quit smoking						**X**	
Ecigs are so freaking cool and now I have one!							**X**

Each X and colored cell indicates the example tweet would have been classified as indicating the corresponding reason.

All analyses relied on anonymous data and adhere to the terms and conditions, terms of use, and privacy policies of Twitter. To protect the privacy of the study participants no tweets were quoted in this study. Instead, example tweets are shown to be indicative of the types of tweets included in our analysis. All analyses were computed using R Ver. 3.2.2.

## Results

**Fig 1:**
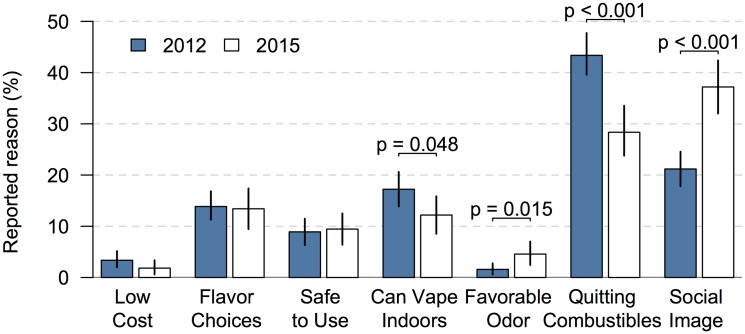
Reasons for vaping (using electronic nicotine delivery systems) inferred from public Twitter postings, 2012–2015. Each bar shows the percentage of reason-related tweets for the focal reason by year with bootstrapped 95% confidence intervals and non-parametric chi-squared test results between years shown where statistically significant.

During 2012 quitting combustibles was the most cited reason for using ENDS. Forty-three percent 43% (95%CI 39–48) of reason-related-tweets mentioned quitting combustibles, *e*.*g*., *“I couldn’t quit till I tried ecigs”*, eclipsing all other reasons by more than double (Fig 1).

The remaining reasons fell into 3 strata by rank. Ranking second, third, and fourth, and significantly higher than all remaining reasons was social image which was cited in 21% (95%CI 18–25) of reason-related-tweets, *e*.*g*., *“I want one of those e-cigs*, *it'll make me look cool*.*”* use indoors, e.g., “*vaping in the club”* which was cited in 17% (95%CI 14–20) of reason-related tweets, and flavor choices, *e*.*g*., “…the best part is the taste,” which was cited in 14% (95%CI 11–17) of reason-related tweets. Ranking fifth and significantly higher than all remaining reasons, safety relative to combustibles, *e*.*g*., “…and vaping is safe,” was cited in 9% (95%CI 7–11) of reason-related tweets. The final strata included ENDS’ favorable odor and low cost ranking sixth and seventh with 3% (95%CI 2–5) and 2% (95%CI 1–3), respectively.

By 2015 both quitting combustibles and use indoors significantly declined to 29% (95%CI 24–33) and 12% (95%CI 9–16) falling from first to second in the rankings and third to fourth in the rankings. At the same time, social image increased to 37% (95%CI 32–43) and favorable odor increased to 5% (95%CI 2–5), the former eclipsing all reasons in 2015 and the latter rising from seventh to sixth in the ranking of reasons reported on Twitter. Other reasons remained stable from 2012 to 2015.

## Discussion

Without any priming or direct costs associated with data collection, public health can use social media surveillance to understand why people vape, yielding actionable intelligence for decision making regarding ENDS now and a pathway forward for additional intelligence using our novel strategy in the future.

Our findings regarding 2012 confirmed with traditional studies; including using ENDS to quit combustibles and use indoors [22,23]. But by 2015 the reasons for vaping as reported on Twitter shifted, with both quitting combustibles and vaping indoors declining in mentions, a finding that has not been reported elsewhere. Taken together with other more recent findings and anecdotal evidence, the shift in reported reasons for vaping appears face valid. Google searches for ENDS for quitting smoking have been on the decline [2] supporting our conclusion that cessation is declining as a reason for vaping. Nearly 500 legislative bodies now ban vaping where smoking is prohibited [24] meaning ENDS cannot be used to avoid clean air laws as before. Similarly ENDS marketing has substantially grown with a focus on social image [25–27] consequently this may be why positive social image dominated all reported reasons on Twitter in 2015 [28].

At the same time, our findings suggest the reasons for using ENDS is in part to circumvent existing policy regulations for controlling combustible tobacco use. Three of the 7 most cited reasons for vaping focused on evading policies, such as novel flavors (*e*.*g*., cherry) that have been banned in cigarettes [29]. The appeal of ENDS can potentially be curtailed by targeting these reasons with public policies that ban the use of non-tobacco flavors in ENDS, apply clean indoor air laws to ENDS, and tax ENDS like combustible tobacco products. Moreover, given these reasons were cited in about 30% of tweets during 2015 a change in public policy could have substantial impact on the appeal of ENDS.

The most important implication is the long-term value of our strategy for open-ended and real-time surveillance, and how it resembles a massive and passive focus group. For example, assuming 2% of adults use ENDS and a sample of 1,000 participants using random-digit dialing costs roughly $70,000, 50,000 interviews would need to be completed to have a single comparable snapshot—a fiftyfold increase in cost representing about $3.5 million. This does not mean our approach replaces surveys, but it can inform their design so that surveys are asking “the right” questions about reasons that are known to resonate with the public and public health leaders have an agile and replicable early surveillance system to guide debate.

Big data has already played a central role in ENDS surveillance, [2,12,30,31] even first identifying their popularity explosion [1], and has similarly improved tobacco control surveillance generally [32–38]. Yet, most studies using Twitter are superficial, describing a general trend for keyword searches like “cholera” [39] or “quit smoking” [40], as detailed in several recent critiques [41–44]. Herein we deviate from the existing literature and demonstrate a protocol that renders specific data for a specific, but poorly addressed, high priority research question that takes advantage of the richness in Twitter data by going beyond keyword-based analyses. Moreover, our strategy has implications beyond ENDS or tobacco control, holding value for studying the appeal of medical devices, public policies, illicit drugs, and commercial products.

In our demonstrative study, we constrained our focus to the most common reasons and analyzed all tweets in aggregate, but in the future this procedure can be refined to overcome these limitations. For example, our strategy can discover additional reasons or variations within reasons *(e*.*g*., what are the most popular flavors?). Moreover, reasons can be described across demographic traits that can be inferred from a Twitter profile, such as gender, ethnicity, and location, yielding demographically specific insights akin to the traditional survey. This potential along with the empirical insights herein suggests our protocol holds great value for ENDS, tobacco control, and public health surveillance going forward.
